# Expansion of a superconducting vortex core into a diffusive metal

**DOI:** 10.1038/s41467-018-04582-1

**Published:** 2018-06-11

**Authors:** Vasily S. Stolyarov, Tristan Cren, Christophe Brun, Igor A. Golovchanskiy, Olga V. Skryabina, Daniil I. Kasatonov, Mikhail M. Khapaev, Mikhail Yu. Kupriyanov, Alexander A. Golubov, Dimitri Roditchev

**Affiliations:** 1 Moscow Institute of Physics and Technology, 141700 Dolgoprudny, Russia; 2 Institut des Nanosciences de Paris, Sorbonne Université, CNRS, UMR7588, 75251 Paris, France; 3 Institute of Solid State Physics RAS, 142432 Chernogolovka, Russia; 4 Fundamental Physical and Chemical Engineering Department, MSU, 119991 Moscow, Russia; 5 National University of Science and Technology MISIS, 119049 Moscow, Russia; 6 Faculty of Computational Mathematics and Cybernetics MSU, 119991 Moscow, Russia; 7 Skobeltsyn Institute of Nuclear Physics, MSU, 119991 Moscow, Russia; 8 Solid State Physics Department, Kazan Federal University, 420008 Kazan, Russia; 9 Faculty of Science and Technology and MESA+ Institute of Nanotechnology, 7500 AE Enschede, The Netherlands; 10 LPEM, ESPCI Paris, PSL Research University, CNRS, 75005 Paris, France; 11 Sorbonne Université, CNRS, LPEM, 75005 Paris, France

## Abstract

Vortices in quantum condensates exist owing to a macroscopic phase coherence. Here we show, both experimentally and theoretically, that a quantum vortex with a well-defined core can exist in a rather thick normal metal, proximized with a superconductor. Using scanning tunneling spectroscopy we reveal a proximity vortex lattice at the surface of 50 nm—thick Cu-layer deposited on Nb. We demonstrate that these vortices have regular round cores in the centers of which the proximity minigap vanishes. The cores are found to be significantly larger than the Abrikosov vortex cores in Nb, which is related to the effective coherence length in the proximity region. We develop a theoretical approach that provides a fully self-consistent picture of the evolution of the vortex with the distance from Cu/Nb interface, the interface impedance, applied magnetic field, and temperature. Our work opens a way for the accurate tuning of the superconducting properties of quantum hybrids.

## Abstract (web summary)

Quantum condensates may penetrate from one material to another due to the proximity effect. Here, Stolyarov et al. report the spatial evolution of quantum vortices from a superconducting Nb layer to a 50 nanometer thick diffusive metallic Cu-film, which is quite thick away from the interface.

## Introduction

The proximity effect occurs at interfaces between superconducting (S) and normal (N) metals. It is due to the penetration of superconducting correlations into the N material and of non-superconducting quasiparticles into the superconductor^1–3^. As a result, the N-layer acquires a relatively long-range quantum coherence from the superconductor, while on the S side the superconducting order parameter is partially suppressed. Owing its quantum coherent nature, the proximity effect is a key ingredient for operation of various superconducting quantum coherent devices, ranging from simple Josephson junctions to quantum computers^4–12^.

In the vicinity of the S/N interface the proximity effect leads to specific spectral features in the quasiparticle excitation spectrum^13–15^. In the diffusive regime and at a finite thickness *d*_N_ of the N-layer, the induced coherent state in N-layer is characterized by an energy gap *δ*, commonly referred to as minigap, which is of the order of the Thouless energy $\sim \hbar D_{\mathrm{N}}{\mathrm{/}}d_{\mathrm{S}}^2$, smaller than the bulk superconducting gap *Δ* in S^16–21^ (see also Methods, subsection Proximity phenomena in the diffusive limit).

Local spectral signatures of the proximity effect were studied in numerous theoretical and experimental works, though, on a microscopic scale, the coherent character of the proximity phenomena was revealed only recently^20,22^. In these works lateral proximity junctions were built, specific phase portraits were produced by applying an external magnetic field, and the quantum coherence was demonstrated through its effect on local spectral signatures in one (1D)^20^ and two dimensions (2D)^22^.

A more general yet complex is the 3D case of a superconducting vortex crossing a S/N interface. From a fundamental point of view, the interest to this problem is related to the special feature of the proximity-induced state, namely that the pair potential *Δ* vanishes on the N side of the S/N bilayer, while the minigap *δ* persists. Therefore, an important question is how the superconducting vortex structure is reproduced in the normal metal. Previous studies^23–26^ were restricted to the limit of very small N layer thickness, at which the proximity vortex essentially mimics the superconducting one. How does the vortex evolve in a thick N-layer? Do these vortices have cores, like Abrikosov vortex in superconductors? What does fix the core size? Both experimental and theoretical understandings of the problem are missing.

**Fig. 1:**
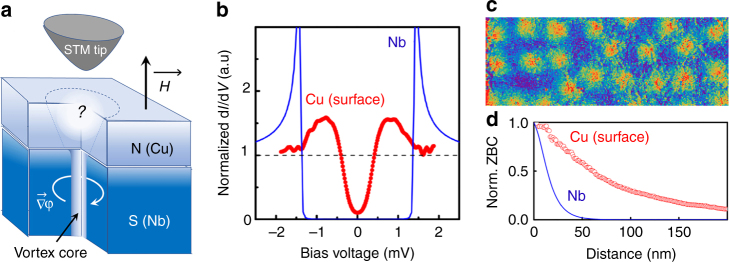
Scanning tunneling spectroscopy experiment. **a** Local tunneling characteristics are probed at the surface of 50 nm thick Cu-film backed with a 100 nm-thick Nb. Superconducting vortices are created in Nb by applying an external magnetic field perpendicularly to Cu/Nb interface; **b** Red data points: tunneling conductance d*I*(*V*)/d*V* spectrum measured at Cu-surface exhibits a minigap *δ*_Cu_ ≃ 0.5 meV; it is three times smaller than the superconducting gap *Δ*_Nb_ ≃ 1.4 meV in the d*I*(*V*)/d*V* spectrum of Nb (blue line); the observed excitations inside the minigap are due to a non-zero residual magnetic field (see the discussion in the main text); **c** 800 nm × 250 nm color-coded ZBC d*I*/d*V*(*V* = 0) map acquired in the magnetic field of 120 mT reveals proximity vortices; **d** Radial variation of the ZBC from the vortex center defines the vortex core profile (red data points). The minigap vanishes in the vortex cores; blue line-expected radial ZBC evolution at the Abrikosov vortex core in Nb-film

In this paper we study the spatial evolution of quantum vortices induced into a diffusive 50 nm thick metallic Cu-film by proximity with a superconducting 100 nm thick Nb layer. The geometry of the experiment is presented in Fig. 1a (see also Methods, subsections Sample preparation and characterization and scanning tunneling spectroscopy experiments). At zero magnetic field a spatially homogeneous proximity minigap is revealed at the surface of Cu-film by scanning tunneling spectroscopy (STS), Fig. 1b. By applying a magnetic field perpendicular to Cu/Nb interface we created Abrikosov vortices in Nb and measured their effect on the tunneling Local Density of States (tunneling LDOS) at Cu-surface. STS maps revealed a disordered lattice of proximity vortices, with a proximity minigap vanishing inside the vortex cores (Fig. 1c, d). Since quantum vortices are direct consequence of the 2*π*-singularity of the macroscopic phase, our STS experiments directly demonstrate that in the diffusive normal Cu the quantum coherence is preserved several tens of nanometers away from the S/N interface. To extend our knowledge about the proximity vortices inside N-electrode we developed a self-consistent theoretical model based on quasi-3D Usadel formalism^27^. A combination of this theory with the results of the surface-sensitive STS experiment offers a complete microscopic picture of the spatial and spectral evolution of the proximity vortex cores in a diffusive metal. Among possible candidates for S/N bilayers we chose Nb/Cu, a system commonly used for SNS junctions^10,28–32^.

## Results

### Experiment

Despite the expected granular structure of sputtered Cu-films^33^ (see also Supplementary Fig. 3a), the tunneling spectra acquired at zero magnetic field are spatially homogeneous; at 300 mK they have a typical shape of a proximity LDOS. The spectra are characterized by broad quasiparticle peaks and a well-developed proximity minigap *δ*_Cu_ ≃ 0.5 meV, which is significantly smaller than the superconducting gap of the underlying Nb, *Δ*_Nb_ = 1.4 meV (Fig. 1b). When the temperature increases, the minigap rapidly vanishes; at *T* = 4–4.5 K it is not observed anymore (the temperature dependence of the tunneling spectra is available in Supplementary Fig. 2c, e).

**Fig. 2:**
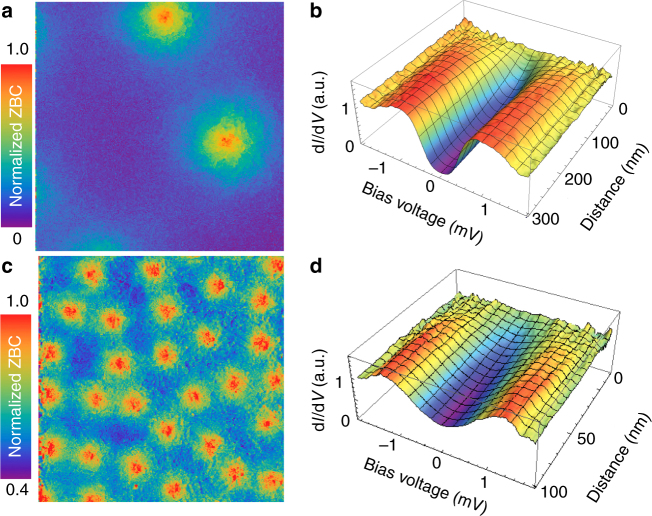
Overlap of proximity vortex cores. **a**, **c** 800 nm × 800 nm ZBC maps acquired at 300 mK in magnetic fields of 5 and 55 mT, respectively. **b**, **d** Radial evolution of the tunneling conductance spectra in the vicinity of the proximity vortex cores. The applied magnetic fields are the same as in **a**, **c**. Due to their large cores, the proximity vortices strongly overlap already at low fields, $H \ll H_{{\mathrm{c2}}}^{{\mathrm{Nb}}} \simeq 4$ T

In contrast, when a magnetic field is applied, the zero-bias conductance (ZBC) maps d*I*/d*V*(*V* = 0) show spatially inhomogeneous distribution of ZBC, forming regular round spots, Fig. 2. As the external magnetic field *H* is increased from 5 to 55 mT, and then to 120 mT, the density of the spots raises in an expected ∝*H* manner (Figs. 1c and 2a, c). In the centers of the spots the minigap vanishes, and a normal state LDOS is recovered (Fig. 2b, d). The spots can therefore be unambiguously interpreted as cores of quantum vortex generated in Nb, crossing the entire Cu-film, and emerging at the surface. The ZBC profiles of these vortices (Fig. 1c) are similar to those of Abrikosov vortex cores in superconductors^24,34–38^. However, they are by a factor of 4 larger than the expected vortex core profile in the underlying superconducting Nb. The understanding of this effect is not trivial, and requires additional theoretical considerations that we present below.

### Theory and numerical method

We performed our theoretical calculations in the framework of the quasiclassical Usadel formalism^27^. The advantage of this approach is that in the diffusive limit, *l* ≪ *ξ* (where *l* and *ξ* are respectively the mean free path and the coherence length), it enables accurate calculations of the spatial and energy variations of the quasiparticle excitation spectra in superconducting hybrids (Usadel fits of experimental tunneling conductance spectra acquired in zero-field are available in Supplementary Fig. 2d, f).

The drawback of the Usadel approach resides in a technical complexity of solving differential Usadel equations self-consistently in spatially inhomogeneous 3D-systems, with properly including the self-consistency and taking into account various boundary conditions. These are nevertheless required to describe realistic superconducting hybrids under magnetic filed. This complexity explains why the Usadel model has mainly been used to solve 1D or quasi-1D problems, rare exceptions being the original works by Cuevas and Bergeret^39,40^ who solved Usadel equations in 2D and predicted the existence of quantum vortices in the proximity area of diffusive SNS junctions, and a recent report by Amundsen and Linder^41^. In the present work we took advantage of a quasi-cylindrical geometry of the vortex core and replaced the hexagonal vortex lattice unit cell by a circular one. This so-called Wigner–Seitz approximation^42^ is reasonable at low fields when the size of the vortex lattice unit cell is significantly larger than the lateral size of the vortex core. Notice, that the Wigner–Seitz approximation we use here to define the coordinate dependence of the Green’s function has been previously successfully used to study the Abrikosov vortex lattice and the influence of trapped Abrikosov vortices on properties of tunnel Josephson junctions^16,43^.

**Fig. 3:**
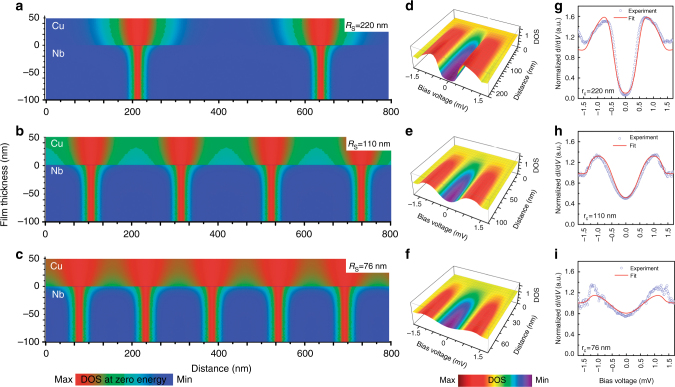
Calculated local DOS inside Cu/Nb-bilayer. **a**–**c** Color-coded zero-bias DOS (*r*, *z*) maps calculated for three different magnetic fields 5, 55, and 120 mT, respectively. **d**–**f** Calculated 3D-plot of the radial evolution of the tunneling DOS in the vicinity of the proximity vortex cores; the magnetic fields are the same as in **a**, **c**. **g–i** Dots: experimental tunneling spectra acquired away from the vortex cores at three magnetic fields as in **a**–**c**—lines: DOS fits calculated at the edge *R*_S_(*H*) of the circular vortex lattice unit cell (see in the text)

We a priori assumed that the conditions of the dirty limit are valid for both S and N films, whose thicknesses are defined respectively as *d*_S_ and *d*_N_. The pair potential, *Δ* is considered zero in N. We aligned the *z*-axis with $\vec H$, and placed the origin at the interface between S and N metals. The S and N layers are therefore located at −*d*_S_ ≤ *z* ≤ 0 and 0 ≤ *z* ≤ *d*_N_, respectively (Fig. 3a–c). According to the Wigner–Seitz approach the hexagonal unit cell of the vortex lattice is replaced by a circular one with a radius *R*_S_ = $R_{\mathrm{c}}\sqrt {H_{{\mathrm{c2}}}{\mathrm{/}}H}$, where the critical radius *R*_c_ = $\sqrt {{ {\Phi }}_{\mathrm{0}}{\mathrm{/}}\pi H_{{\mathrm{c2}}}}$ (*Φ*_0_ is the magnetic flux quantum) and the second critical field *H*_c2_ are determined by the well-known expressions^42^:

1 $${\mathrm{ln}}{\kern 1pt} t + \psi \left( {\frac{1}{2} + \frac{t}{{r_{\mathrm{c}}^2}}} \right) - \psi \left( {\frac{1}{2}} \right) = 0.$$

In Eq. (1) *ψ*(*x*) is the digamma function, *t* = *T*/*T*_c_—reduced temperature, *r*_c_ = *R*_c_/*ξ*_S_ is the reduced critical radius, *ξ*_S_ = $\sqrt {\hbar D_{\mathrm{S}}{\mathrm{/}}2\pi k_{\mathrm{B}}T_{\mathrm{c}}}$, is the effective superconducting coherence length (An often used formula $\xi _{\mathrm{S}} = \sqrt {D_{\mathrm{S}}{ {/\Delta }}}$ differs from our definition only by a numerical factor $\sqrt {2\pi {\mathrm{/1}}{\mathrm{.76}}} \simeq 1.89$), and *D*_S_ is the diffusion coefficient in S.

Under the above assumptions, the system of Usadel equations^27^ describing the behavior of S/N bilayer in a magnetic field in cylindrical (*r*, *z*) coordinates has the form^16,43^:

2 $$\frac{{\mathrm{d}^2\theta _{\mathrm{S}}}}{{{\mathrm{d}}{z}^2}} + \frac{1}{r}\frac{\mathrm{d}}{{{\mathrm{d}}{r}}}\left( {r\frac{{\mathrm{d}\theta _{\mathrm{S}}}}{{\mathrm{d}r}}} \right) - ({ {\Omega }} + Q^2{\mathrm{cos}}{\kern 1pt} \theta _{\mathrm{S}}){\mathrm{sin}}{\kern 1pt} \theta _{\mathrm{S}} = - { {\Delta }}{\kern 1pt} {\mathrm{cos}}{\kern 1pt} \theta _{\mathrm{S}},$$

3 $$\frac{{\mathrm{d}^2\theta _{\mathrm{N}}}}{{{\mathrm{d}}{z}^2}} + \frac{1}{r}\frac{\mathrm{d}}{{{\mathrm{d}}{r}}}\left( {r\frac{{\mathrm{d}\theta _{\mathrm{N}}}}{{{\mathrm{d}}{r}}}} \right) - \frac{{{ {\Omega }} + k^2Q^2{\kern 1pt} {\mathrm{cos}}{\kern 1pt} \theta _{\mathrm{N}}}}{{k^2}}{\mathrm{sin}}{\kern 1pt} \theta _{\mathrm{N}} = 0,$$

4 $$Q = \frac{1}{r}\left( {1 - \frac{{r^2}}{{r_{\mathrm{S}}^2}}} \right),$$

5 $${ {\Delta }}{\kern 1pt} {\mathrm{ln}}{\kern 1pt} t + 2t\mathop {\sum}\limits_{\Omega \ge 0}^\infty \left( {\frac{{\mathrm{\Delta }}}{{ {\Omega }}} - {\mathrm{sin}}{\kern 1pt} \theta _{\mathrm{S}}} \right) = 0.$$

Here *θ*_S(N)_ are complex pairing angles related to the local DOS in S(N) as *N*_S(N)_(*r*, *z*, *ε*) = Re{cos*θ*_S(N)_}, *Ω* = (2*n* + 1)*t* are the Matsubara frequencies, *ξ*_N_ = $\sqrt {\hbar D_{\mathrm{N}}{\mathrm{/}}2\pi k_{\mathrm{B}}T_{\mathrm{c}}}$, *D*_N_ is the diffusion coefficient in N, *k* = *ξ*_N_*/ξ*_S_, *Q* is the circular component of the vector potential **Q** = (0, *Q*, 0) normalized to *Φ*_0_*/*2*πξ*_S_. The pair potential *Δ* in (2)–(5) is normalized to *πk*_B_*T*_c_, and the coordinates *r*, *z* are normalized to *ξ*_S_. The physical meaning of *ξ*_N_ is discussed at the end of the paper.

To write down the solution of the Maxwell equation, ∇ × ∇ × **Q** = *κ*^−2^**J**, for the vector potential **Q** in the form of Eq. (4), we have assumed that the Ginzburg–Landau parameter $\kappa = \lambda _{{\mathrm{S}} \bot }{\mathrm{/}}\xi _{\mathrm{S}} \gg 1$. This condition allows one to neglect the magnetic field produced by supercurrents in comparison with the externally applied field *H*. The external field is therefore considered constant inside a circular vortex cell provided that the reduced cell radius *r*_S_ = R_S_/*ξ*_S_ is smaller than *λ*_S⊥_ = ${\mathrm{max}}(\lambda _{\mathrm{S}},\lambda _{\mathrm{S}}^2{\mathrm{/}}d_{\mathrm{S}})$, where *λ*_S_ is the London penetration depth in S.

Equations (2)–(5) should be supplemented by the boundary conditions^13^ at the S/N interface (*z* = 0):

6 $$\gamma _{\mathrm{B}}k\frac{{\mathrm{d}\theta _{\mathrm{N}}}}{{\mathrm{d}z}} = {\mathrm{sin}}{\kern 1pt} \theta _{\mathrm{N}}{\mathrm{cos}}{\kern 1pt} \theta _{\mathrm{S}} - {\mathrm{sin}}{\kern 1pt} \theta _{\mathrm{S}}{\kern 1pt} {\mathrm{cos}}{\kern 1pt} \theta _{\mathrm{N}},$$

7 $$\frac{{\mathrm{d}\theta _{\mathrm{S}}}}{{\mathrm{d}z}} = \gamma k\frac{{\mathrm{d}\theta _{\mathrm{N}}}}{{\mathrm{d}z}},$$

where *γ*_B_ and *γ* are the suppression parameters

8 $$\gamma _{\mathrm{B}} = \frac{{R_{{\mathrm{SN}}}{\cal A}_{{\mathrm{SN}}}}}{{\rho _{\mathrm{N}}\xi _{\mathrm{N}}}},\quad \gamma = \frac{{\rho _{\mathrm{S}}\xi _{\mathrm{S}}}}{{\rho _{\mathrm{N}}\xi _{\mathrm{N}}}}.$$

Here, *R*_SN_, and, ${\cal A}_{{\mathrm{SN}}}$, are, respectively, the resistance and the area of the S/N interface, *ρ*_S(N)_, are the normal state resistivities of S(N) metals. At the bottom S/Vacuum interface and at the top N/Vacuum interface the boundary conditions has the form:

9 $$\frac{{\mathrm{d}\theta _{\mathrm{S}}}}{{\mathrm{d}z}} = 0,\quad z = - d_{\mathrm{S}},$$

10 $$\frac{{\mathrm{d}\theta _{\mathrm{N}}}}{{\mathrm{d}z}} = 0,\quad z = d_{\mathrm{N}},$$

In addition, at the vortex unit cell border, *r* = *r*_S_, and in its center, *r* = 0, we have, respectively:

11 $$\frac{{\mathrm{d}\theta _{\mathrm{N}}(r_{\mathrm{S}})}}{{\mathrm{d}r}} = \frac{{\mathrm{d}\theta _{\mathrm{S}}(r_{\mathrm{S}})}}{{\mathrm{d}r}} = 0,\quad \theta _{\mathrm{N}}(0) = \theta _{\mathrm{S}}(0) = 0.$$

The boundary value problem (2)–(11) has been solved numerically. Modified Newton method was evaluated to resolve non-linearity of the differential problem (2)–(10). To improve the convergency of Newton method we applied a simple dumping^44^. This continuous Newton procedure brings us to a set of linear differential problems which are solved by applying a special form of the Finite Element Method (FEM), the so-called mixed FEM^45^, which solves simultaneously for both, complex angles *θ*_S_, *θ*_N_, and their gradients. Importantly, the developed FEM form enables to solve problems with discontinuous solutions which may arise from non-standard interface conditions (6), describing a discontinuity of anomalous Green’s functions at the S/N interface. We implemented the whole Newton method—FEM procedure in the framework of finite element package FreeFEM++ (http://www.freefem.org/ff++)^46^.

The complete numerical procedure consists of two stages. It starts with solving the boundary value problem (2)–(11) and with the determination of the spatial dependence of the pair potential *Δ*(*r*). At the second stage, we perform the analytical continuation in the Eqs. (2)–(10) by replacement *ω* → −*iε*, and for the already known *Δ*(*r*) dependence, in order to calculate the dependence of the density of states *N*(*r*, *z*, *ε*) = Re{cos*θ*_N_} on energy *ε* at an arbitrary position *r*, *z*.

### Results of numerical calculations

Using the numerical method described above, we have calculated the spatial evolution of the LDOS near the vortex core in the Nb/Cu-bilayer. The results of the calculations are presented in Fig. 3. Figure 3a–c presents the (*r*, *z*)-spatial evolution of ZB-DOS for three different values of the magnetic field, corresponding to the experimental data presented in Figs. 1 and 2. The validity of this result is confirmed by an excellent agreement between the calculated LDOS profiles in Fig. 3d–f and the experimental ones in Fig. 2b, d, both corresponding to *z* = 50 nm, i.e. to the LDOS at the surface of Cu-film. Figure 3g–i shows some of the calculated tunneling conductance spectra at the surface of the Cu film in between vortices (red lines), and their comparison with the experimental STS data (dots), also demonstrating a nice agreement. Importantly, in these calculations the resistivity *ρ*_Cu_ and the Nb/Cu-interface resistivity *R*_SN_*A*_SN_ were taken as the only fitting parameters. Once adjusted, they were fixed to calculate the excitation spectra for all positions, fields and temperatures.

The results presented in Fig. 3 were all obtained taking *ρ*_Cu_ = 3.7 μΩ cm and *R*_SN_*A*_SN_ = 1.5 × 10^−11^ Ω cm^2^, both values being typical for in situ fabricated Nb/Cu structures^47^. All other parameters of the model were calculated on the basis of these two fitting parameters and well-established relations. The mean free path in the Cu-film, *l*_Cu_ = 18 nm, was determined using the well-known empiric relation (*l*_Cu_*ρ*_Cu_)^−1^ = 1.54 × 10^11^ Ω^−1^ cm^−2^^48^. This *l*_Cu_ value corresponds well to the grain size of our Cu-film estimated from STM images (Supplementary Fig. 3a). The parameter *ξ*_N_ in Cu, *ξ*_Cu_ = 37 nm, was calculated using $D_{{\mathrm{Cu}}} = l_{{\mathrm{Cu}}}v_F^{{\mathrm{Cu}}}{\mathrm{/}}3$, $v_{\mathrm{F}}^{{\mathrm{Cu}}}$ = 1.57 × 10^6^ m/s. The critical temperature of the bilayer, T_c_ = 8.1 K, was extracted from the transport experiment (see Methods, subsection Sample preparation and characterization, and Supplementary Fig. 2a). That fixes, in turn, the proximity parameters, *γ* = 0.53 and *γ*_B_ = 1.1. The coherence length in Nb, *ξ*_Nb_ = 9 nm, was taken from^49^.

## Discussion

We now turn to the problem of the induced quantum coherence in N. The parameter *ξ*_N_ we used in Usadel equations is, by a numerical factor $\sqrt {2\pi {\mathrm{/}}1.76} \simeq 1.89$, the so-called normal coherence length $\sqrt {\hbar D_{\mathrm{N}}{ {/\Delta }}_0}$, which is often associated in literature with the quantum coherence length in N (*Δ*_0_ = 1.76*k*_B_*T*_c_ is zero-temperature gap in S). On the microscopic level however, the proximity phenomena in N are described by Andreev quasiparticles: pairs of coherent electrons and retro-scattered holes which are converted into/from Cooper pairs at the S/N interface (see^15^). The important point here is that if the energy of an Andreev electron with respect to the Fermi energy is *E* (usually, *E* ≤ *Δ*) the hole has the energy −*E*. Quasiclassically, such electron–hole pair dephases in time; a typical dephasing time is *t* ~ *ħ*/*E*. In a diffusive metal, this time is associated with a distance $L_{ {E}} = \sqrt {D_{\mathrm{N}}t} \approx \sqrt {\hbar D_{\mathrm{N}}{\mathrm{/}}E}$. It is immediately clear that for an Andreev quasiparticle of an energy *E* = *Δ* the characteristic dephasing coherence length is indeed *L*_*E*_ = 1.89*ξ*_N_. However, other Andreev quasiparticles, having energies lower than *Δ*, remain coherent over longer distances, *L*_*E*_ > *ξ*_N_. Theoretically, the coherence length could even be infinite, as *L*_*E*_ → ∞ for *E* → 0. In real systems, thermal excitations ~*k*_B_*T* and the Thouless energy $E_{{\mathrm{Th}}} = \hbar D_{\mathrm{N}}{\mathrm{/}}d_{\mathrm{N}}^2$, related to the physical size of N-subsystem, limit the spatial extent of coherent Andreev quasiparticles in N. At low temperatures of our STS experiment, the characteristic energy is the minigap, *δ* > *k*_B_*T*, and the effective coherence length in N should be $L_\delta = \sqrt {\hbar D_{\mathrm{N}}{\mathrm{/}}\delta }$. Putting the value for *δ* obtained both experimentally and theoretically, we get *L*_*δ*_ ≈ 112 nm, i.e., *L*_*δ*_ ≠ *ξ*_N_^15^.

The parameter *ξ*_N_ rather defines a region near S/N interface where the DOS strongly evolves from a BCS-like to the minigap. Far enough from this transition region, the proximity DOS should not evolve strongly^15^. Indeed, by analyzing theoretical evolution of the vortex core inside N (follow, for instance, the color plot in Fig. 3a) it becomes clear that after a jump at S/N interface, and a rapid evolution in N over 30–50 nm, the vortex core size indeed tends to a saturation at the surface. Therefore, the lateral size of the proximity vortex core measured at the surface by STS should give a good estimate for the effective coherence length in N.

**Fig. 4:**
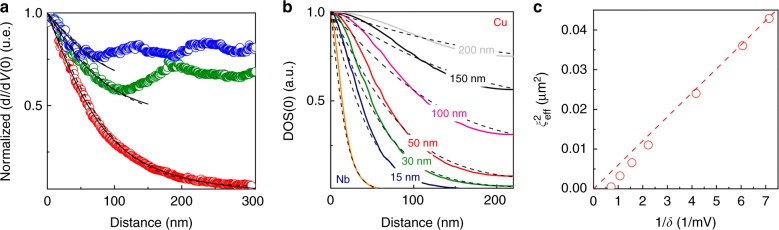
Extracting the effective coherence length in Cu. **a** Red, green and blue data points: radial ZBC profiles of proximity vortex measured in magnetic fields of 5, 55, and 120 mT, respectively. Dashed lines: fits using phenomenological formula suggested in ref. ^51^; Solid lines: fits using modified de Gennes’ approach developed in ref. ^50^. Both models fit well the experimental with *ξ*_eff_ = 105–110 nm (see in the text). **b** Color lines: vortex core profiles (zero-bias conductance) calculated within Usadel framework for different thicknesses of Cu-film in 5 mT field. Dashed lines—best fits using the approximate formula suggested in^51^ (see in the text). **c** The *ξ*_eff_ vs *δ* plot presented in $\xi _{{\mathrm{eff}}}^2 - 1{\mathrm{/}}\delta$ coordinates

The measured ZBC vortex core profiles are presented as color circles in Fig. 4a. Solid lines are the fits using the approach developed to fit the vortex cores in superconductors in high magnetic fields^50^. The fits are obtained with *ξ*_eff_ = 109 nm. Dashed lines are fits using the phenomenological formula for vortices in superconductors suggested in^51^, *σ*_*H*_(*r*) = 1 − (1 − *σ*_0_) tanh(*r*/*ξ*_eff_), in which *σ*_0_ is the normalized ZBC measured far from the core, and *ξ*_eff_ is the effective coherence length. This empiric formula is commonly used in the STM/STS community to extract the superconducting coherence length from the vortex core profile. The best fit is obtained with *ξ*_eff_ = 105 nm, i.e., indeed *ξ*_eff_ ≈ *L*_*δ*_ > *ξ*_N_. In Fig. 4b we show several vortex core profiles (zero-bias conductance) calculated for different N-thicknesses and compare them to fits by the above formula for *σ*_*H*_(*r*). One can see that the formula works well for the S system alone and qualitatively reproduces the overall dependence for S/N bi-layers. By evaluating *ξ*_eff_ and *δ* for samples with different Cu-thickness we found a nearly linear $\xi _{{\mathrm{eff}}}^2(1/\delta )_{}^{}$ dependence, Fig. 4c, as expected for *L*_*δ*_(*δ*). Therefore, the proximity vortex have indeed a characteristic lateral extension *ξ*_eff_ ≈ *L*_*δ*_. A simplified picture here is that at distances >*ξ*_N_ from S/N interface, the proximity vortex cores look like the cores of Abrikosov vortices in superconductors. Consequently, the N-electrode of a finite thickness $d_{\mathrm{N}} < \sqrt {\hbar D_{\mathrm{N}}{\mathrm{/}}k_{\mathrm{B}}T}$ can be thought as a genuine superconductor with *δ* and *L*_*δ*_ playing respective roles of the effective superconducting gap and coherence length.

The round shape and well-defined size ~ 2*L*_*δ*_ of the observed proximity vortex cores make them substantially different from the Josephson vortices predicted in^39^ and recently observed in N-parts of lateral SNS junctions^22^. In these works, the Josephson vortex cores were found distorted. Their width is fixed by the width of the N-part of the SNS junction, whereas their length along S/N interface varies with the applied magnetic field. The length is defined by a typical distance over which the quantum phase along each S/N interface accumulates a *π*-shift. In rising magnetic field, the phase gradients along S/N interfaces increase, and the length of the Josephson proximity vortex cores decreases. The minimum length of thermodynamically stable Josephson vortex cores is limited by critical currents at S-edges to ~*ξ*_S_. Up to now both kinds of vortices were observed only in the diffusive regime. Extending experimental studies to the ballistic limit^52^ is a challenging task for the future.

**Fig. 5:**
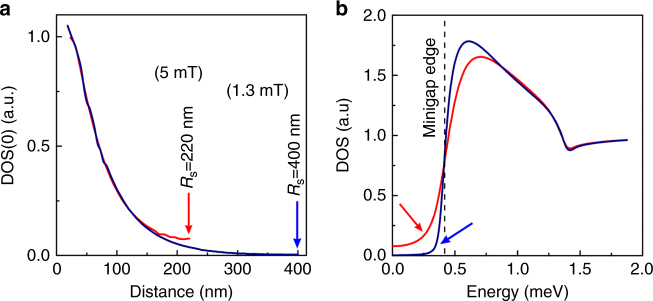
Quasiparticle excitations inside the minigap. **a** Radial ZB-DOS vortex profile for the 50 nm Cu-thick sample in the field 1.3 mT (blue line) and 5 mT (red line). The profiles differ significantly at the vortex periphery. **b** Calculated LDOS at the vortex lattice unit cell boundary for the fields 1.3 mT (blue curve) and 5 mT (red curve). The minigap filling with quasiparticle states is evidenced by arrows

The variation of *L*_*δ*_ and *δ* with the N-layer thickness, as well as the high precision of the Usadel approach enable engineering S/N bi-layers with desired properties (see also Methods, subsection Numerical calculations). The critical temperature, currents and fields can be tuned, providing a route for new functionalities^11^. The minigap filling with quasiparticle excitations due to circulating currents can also be optimized—an important point for engineering superconducting qubits, in studies of Majorana states, Shiba bands, topological superconductivity. Precisely, we found that the density of quasiparticle excitations inside the minigap strongly depends on magnetic field (via orbital effect) and on N-layer thickness. Figure 5a presents ZB-DOS vortex profiles at 1.3 and 5 mT in the 50 nm Cu-thick sample. At 1.3 mT (*R*_*S*_ = 400 nm), the minigap continues to exist at the vortex lattice unit cell boundary, as ZB-DOS is zero there. However, already at 5 mT (*R*_S_ = 220 nm) the minigap transforms to a pseudo-minigap, ZB-DOS > 0. In Fig. 5b the LDOS at the unit-cell boundary (*r* = *R*_S_) is plotted as a function of energy, for the same fields. It is immediately clear that when the the radius *R*_*S*_ of the vortex unit cell becomes comparable or smaller than *L*_*δ*_, the minigap fills with quasiparticle excitations (even between vortices). The phenomenon takes place first at the minigap edges and extends to all in-gap states, Fig. 5b. In Fig. 4b we plot radial ZB-DOS profiles for samples of different Cu-thickness. They demonstrate that as Cu-thickness is increased and the zero-field minigap becomes smaller and smaller, the density of in-gap quasiparticle excitations rapidly increases, thus transforming the hard minigap in a sort of a pseudo-minigap. This demonstrates the fragility of the minigap with respect to circulating currents, and puts constraints for applications.

In conclusion, we experimentally and theoretically demonstrated the existence of a well-defined core of quantum vortex induced from a superconductor (Nb) into a diffusive normal metal (Cu). By mapping the spatial variations of the proximity minigap in the local tunneling spectra, we measured the core size, and followed the evolution of the cores with temperature and magnetic field. We complemented our observations by a self-consistent model based on quasi-classical Usadel approach. We developed a numerical method that allowed us to calculate with high precision the quasiparticle excitation spectra near the vortex cores at realistic conditions of the scanning tunneling spectroscopy experiment, and to discover characteristic spatial and energy scales which are in play inside S/N bilayers. Our results extend the microscopic knowledge about quantum vortex, and enable extracting relevant physical properties of the buried S/N interface that control the proximity phenomena.

## Methods

### Proximity phenomena in the diffusive limit

To describe proximity effect in diffusive superconducting and normal materials, the most complete theoretical framework is provided by the quasiclassical theory of superconductivity based on Usadel equations^16,18,19,27,53^. A general hallmark found for a SN bilayer is that the superconducting correlations induced in N probed at energy *E* remain coherent over a length $L_{ {E}} = \sqrt {\hbar D_{\mathrm{N}}{\mathrm{/}}E}$, *D*_N_ being the diffusion coefficient in N. This makes naturally appear the coherence length *ξ*_N_ in N associated with the superconducting energy gap *Δ* in S, $\xi _{\mathrm{N}} = \sqrt {\hbar D_{\mathrm{N}}{\mathrm{/\Delta }}}$^16–18^. However an important difference should be made when the thickness *d*_N_ of the N part is finite or infinite. When the thickness is finite a characteristic true energy gap appears commonly referred to as minigap, which is directly linked directly to the Thouless energy of the N part $E_{{\mathrm{Th}}} = \hbar D_{\mathrm{N}}{\mathrm{/}}d_{\mathrm{N}}^2$ in the long junction limit $d_{\mathrm{N}} \gg \xi _{\mathrm{N}}$. On the other hand, for infinite thickness there is no more any energy gap and for any energy *E* the superconducting correlations in N decay over *L*_*E*_ provided that *L*_*E*_ < *L*_*Φ*_, *L*_*Φ*_ being the electronic phase coherence length of N (ref. ^54^, where Eq. (28) explicitly shows $\sqrt E$ dependence of DOS for the case of infinite thickness of N-layer).

Local spectral signatures of the proximity effect were experimentally probed by tunneling for SN junctions with an infinite N system^21,55^, and a finite N system^56,57^ and have thoroughly confirmed the theoretical predictions of the quasiclassical theory. Other important geometries such as Josephson SNS junctions have also been addressed theoretically^13,39,58–61^. Recent experimental studies of SNS junctions^20,22^ further confirmed the robustness of the Usadel theory in the diffusive limit.

### Sample preparation and characterization

The Cu/Nb-bilayers were elaborated using the two-step inverted growth/cleavage method, initially suggested by Karapetrov et al.^62,63^ and Stolyarov et al.^33^. The method enables the preparation, under ultrahigh vacuum, of a large variety of complex hybrid systems with clean exposed surfaces. The latter condition is mandatory for reliable scanning tunneling spectroscopy measurements with high spatial and energy resolution.

The Nb/Cu bilayers were first grown at a base pressure of 5 × 10^−7^ mbar by magnetron sputtering onto a SiO_2_(270 nm)/Si(0.3 mm) wafer kept at room temperature. First, a 50 nm thick film of Cu was deposited on SiO_2_, followed by the deposition of 100 nm of Nb. The SiO_2_ layer is essential to avoid a chemical bonding between Cu and Si, thus preventing a strong mechanical adhesion of the Cu-layer to the substrate (Supplementary Fig. 1a).

Macroscopic superconducting properties of Cu/Nb-bilayers were measured in 4-probe low temperature transport experiments. A sharp transition to a superconducting state was detected at *T*_c_ = 8.1 K, as demonstrated in Supplementary Fig. 2a.

### Scanning tunneling spectroscopy experiments

At the second stage, the samples were glued in air using a UHV-compatible conductive epoxy (Epotek H27D (http://www.epotek.com)), Supplementary Fig. 1. The Nb-side of the sample was glued onto the stainless steel STM sample holder; a cleaver was glued onto the opposite (Si) face of the sandwich (see Supplementary Fig. 1b–d). The sandwiches prepared in this way were introduced into the UHV STM chamber (base pressure of 3 × 10^−11^ mbar). By softly pushing on the cleaver with the help of a manipulator (Supplementary Fig. 1e, f), the Nb/Cu/SiO_2_/Si multilayer structure breaks at the Cu/SiO_2_ interface, the weakest part of the sandwich. The obtained sample, a Cu/Nb-bilayer with Cu as top layer (Supplementary Fig. 1a), was then put into UHV-STM head (Supplementary Fig. 1g). In-situ STM/STS experiments were carried out in the temperature range 0.3–5 K^64^; mechanically etched Pt(80%)/Ir(20%) tips were used. Topographic STM images were obtained in a constant-current mode; STS was realized by acquiring local *I*(*V*)(*x*, *y*) characteristics and numerically differentiating them to obtain the tunneling conductance d*I*/d*V*(*V*)(*x*, *y*) maps.

### Numerical calculations

The details of the numerical method developed within the Usadel model are presented in the Main Text. As a validity check, the method was applied to reproduce the experimentally measured temperature dependence of the tunneling proximity spectra (Supplementary Fig. 2b–f). The temperature evolution of the Zero-Bias Conductance and their fits by Usadel model are presented in Supplementary Fig. 2b. The temperature dependence of the tunneling spectra and their fits are presented in Supplementary Fig. 2c, d. The method gives a detailed agreement with the experimental data, Supplementary Fig. 2e, f. Remarkably, all the fits are generated with the same set of parameters (see the discussion in the Main Text).

Supplementary Figure 3c represents the calculated tunneling DOS of Nb (yellow line) along with the calculated proximity DOS at Cu-surface for various thickness of Cu-layer (15 nm, 30 nm, 50 nm, 100 nm, 150 nm, 200 nm). As the thickness of N-layer increases, the proximity minigap *δ* decreases. The calculated DOS at the Cu-surface for different Cu-film thicknesses enables estimating the proximity minigap.

The developed numerical method enables predicting the evolution of the DOS in the vicinity of the vortex singularity. Supplementary Figure 3b demonstrates the calculated evolution of the vortex core inside the N-layer for different Cu-layer thicknesses at a magnetic field of 5 mT. For small thicknesses the vortex core mimics the core of the Abrikosov vortex in Nb. As the thickness increases, the vortex core rapidly expands in the proximity region. Also important, the jump in the core size at the S/N interface strongly depends on N-layer thickness. Remarkably, the depth-dynamics of the vortex core expansion is slower near the surface, as it is clear for all thicknesses up to 100 nm. These calculations demonstrate the crucial importance of the finite thickness of Cu-layer for the vortex core shape and expansion.

For thicker N-layers (150 nm, 200 nm) the vortex cores rapidly expand to the limit of the vortex unit cell. The situation corresponds to the overlap of the proximity vortex cores which occurs even at low fields of a few mT. This shows how the superconducting correlations are affected by magnetic fields and super-currents; it enables one to tune the magnetic field response of S/N-bilayers.

Supplementary Figure 3d presents the comparison between the vortex core profiles calculated in the framework of Usadel approach and the fits using the approximate formula suggested in^51^ (see also in the Main text). The fits enable to extract the effective coherence length *ξ*_eff_ in the normal layer for different thicknesses of Cu-film.

### Data availability

Authors can confirm that all relevant data are included in the paper and its Supplementary Information files. Additional data are available on request from the authors.

## Electronic supplementary material

Supplementary Information
