# Supplementary material for: Expansion of a superconducting vortex core into a diffusive metal
Source: Nat Commun. 2018 Jun 11;9:2277. doi: 10.1038/s41467-018-04582-1 (PMC5995889; doi:10.1038/s41467-018-04582-1)
Supplement: Supplementary file 1 — Supplementary Information [file 41467_2018_4582_MOESM1_ESM.pdf]

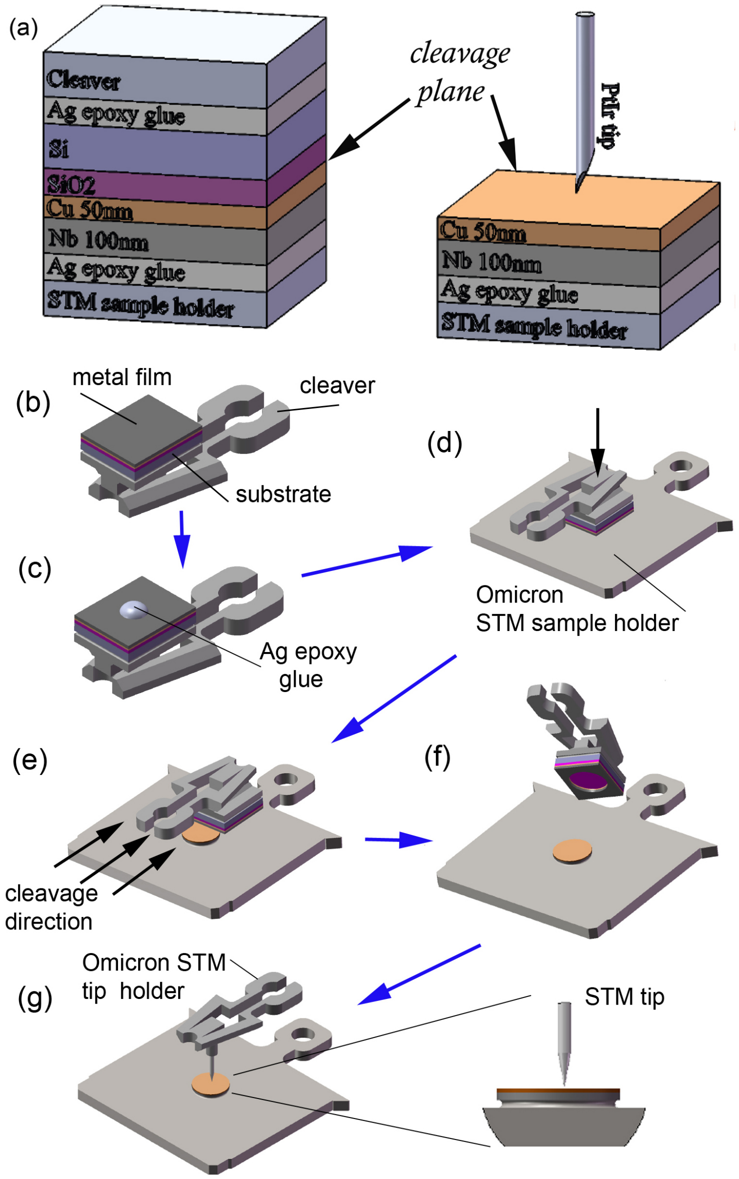

Supplementary Figure 1: Sample preparation. (a) - a schematic representation of the sample before and after cleaving and STM tip orientation; (b-g) - schematic cleaving process.

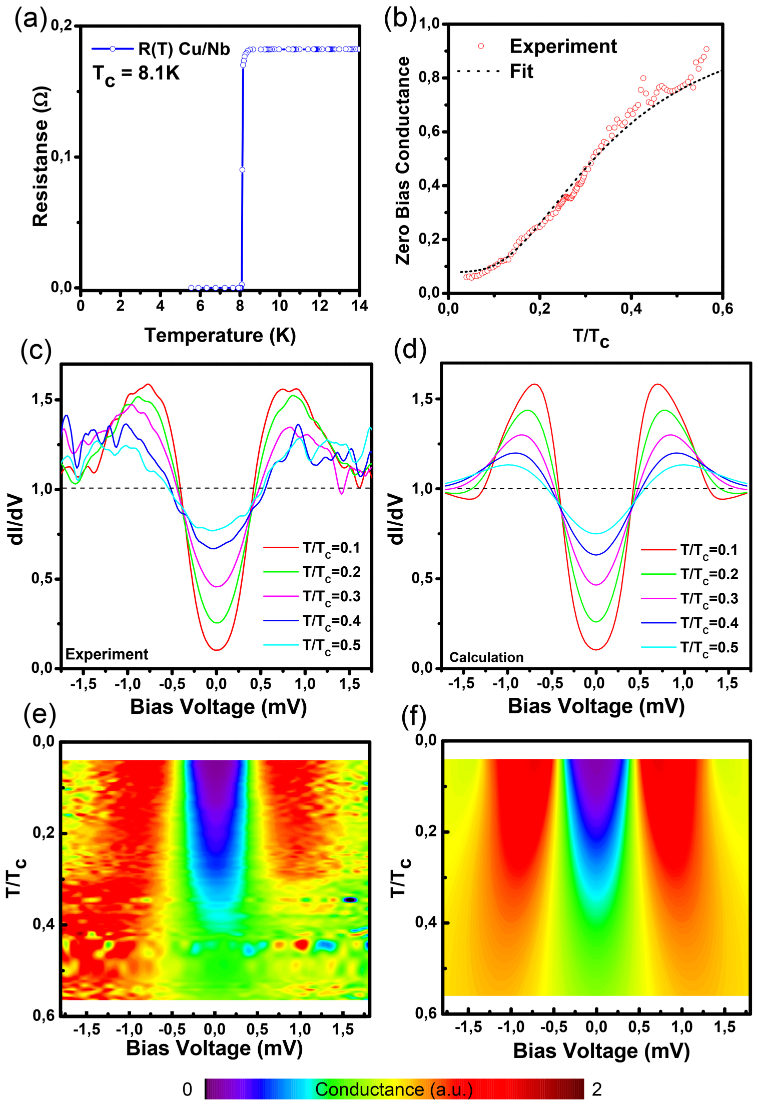

Supplementary Figure 2: Sample characterization and minigap temperature dependance. (a) - resistive transition of the studied Nb/Cu bilayer to a superconducting state; (b) - dots: temperature evolution of the zero-bias tunneling conductance measured by STM/STS at Cu-surface; dashed line - fit by Usadel theory: (c) - experimental dI/dV spectra measured at different temperatures; (d) - best fits of the data in (c) using Usadel model; (e) and (f) - the same as in (c) and (d) but presented as continuous color-coded plots.

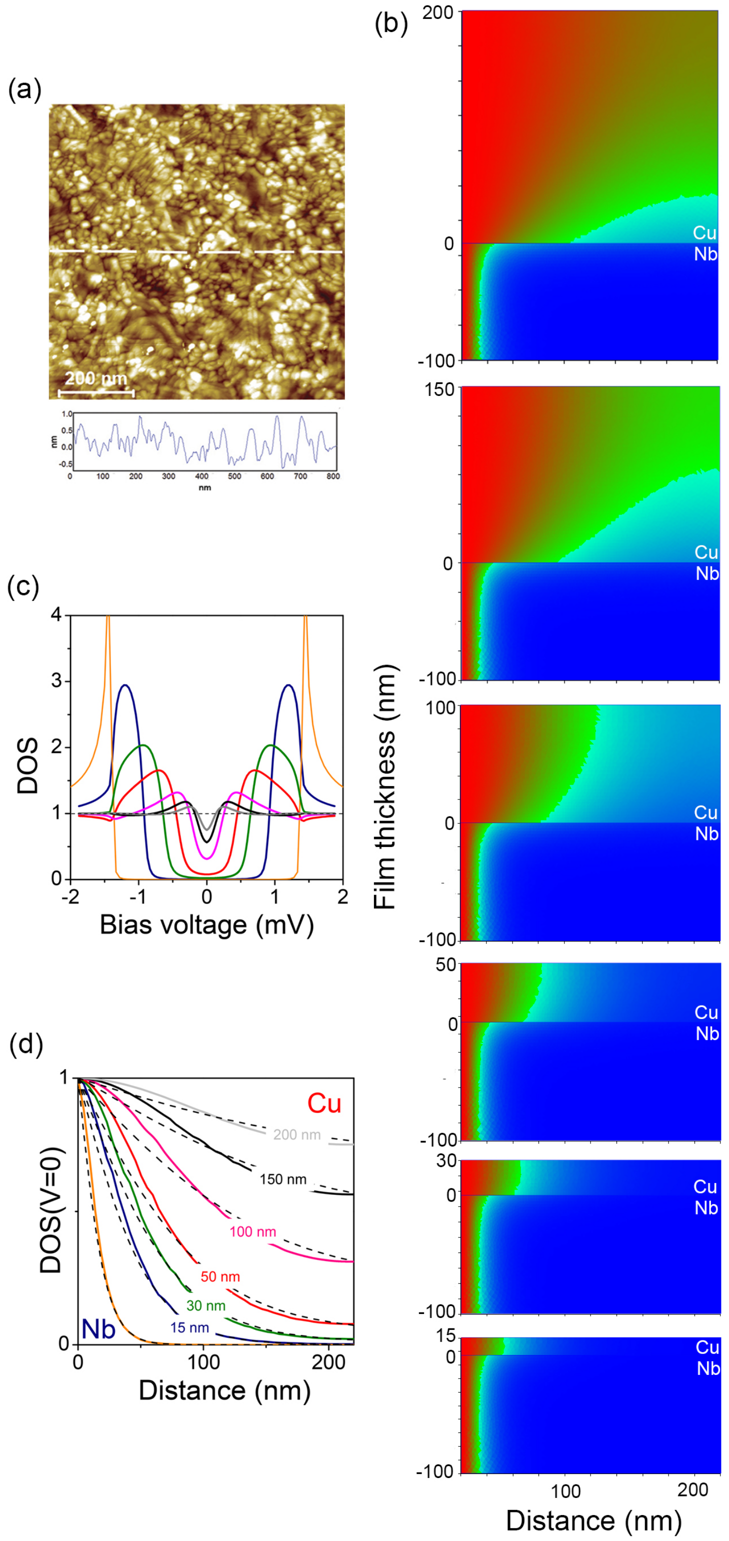

Supplementary Figure 3: STM topography and extended DOS calculations. (a) - 800 nm×800 nm STM topographic image (I= 0.1 nA, sample bias -1 V) with a cross-section plot (the position corresponds to the horizontal white line on the image). (b) - color-coded zero-bias DOS maps of the vortex cores calculated for the fixed field of 5 mT for different thicknesses of Cu-layer (from top to bottom: 200 nm, 150 nm, 100 nm, 50 nm, 30 nm, 15 nm). The color scale is the same as in Fig.3 of the Main Text. (c) - calculated DOS at the Cu-surface for different Cu-film thicknesses allows one estimating the proximity gap δ. (d) - Color lines: vortex core profiles (zero-bias conductance) calculated within Usadel framework for different thicknesses of Cu-film. Dashed lines - best fits using the approximate formula (see in the Main text).
